# Glutathione S-Transferases Play a Crucial Role in Mitochondrial Function, Plasma Membrane Stability and Oxidative Regulation of Mammalian Sperm

**DOI:** 10.3390/antiox9020100

**Published:** 2020-01-24

**Authors:** Marc Llavanera, Ariadna Delgado-Bermúdez, Samuel Olives, Yentel Mateo-Otero, Sandra Recuero, Sergi Bonet, Beatriz Fernández-Fuertes, Marc Yeste, Isabel Barranco

**Affiliations:** 1 Biotechnology of Animal and Human Reproduction (TechnoSperm), Institute of Food and Agricultural Technology, University of Girona, E-17003 Girona Spain; marc.llavanera@udg.edu (M.L.); ariadna.delgado@udg.edu (A.D.-B.); yentel.mateo@udg.edu (Y.M.-O.); sandra.recuero@udg.edu (S.R.); sergi.bonet@udg.edu (S.B.); beatriz.fernandez@udg.edu (B.F.-F.); 2 Unit of Cell Biology, Department of Biology, Faculty of Sciences, University of Girona, E-17003 Girona, Spain; samolives04@gmail.com

**Keywords:** Mammalian sperm, Ethacrynic acid, GSTs, GSTM3, Boar semen, Liquid-storage

## Abstract

Glutathione S-transferases (GSTs) are essential sperm antioxidant enzymes involved in cell protection against oxidative stress and toxic chemicals, preserving sperm function and fertilising ability. Artificial insemination (AI) in pigs is commonly carried out through the use of liquid-stored semen at 17 °C, which not only reduces sperm metabolic activity but also sperm quality and AI-farrowing rates within the 72 h of storage. While one may reasonably suggest that such enzymes are implicated in the physiology and maintenance of mammalian sperm function during liquid-storage, no previous studies conducted on any species have addressed this hypothesis. Therefore, the objective of the present work was to characterise the presence and function of sperm GSTs in mammalian sperm, using the pig as a model. In this regard, inhibition of such enzymes by ethacrynic acid (EA) during semen storage at 17 °C was performed to evaluate the effects of GSTs in liquid-preserved boar sperm by flow cytometry, immunofluorescence, and immunoblotting analysis. The results of this study have shown, for the first time in mammalian species, that the inhibition of GSTs reduces sperm quality and functionality parameters during their storage at 17 °C. These findings highlight the key role of such enzymes, especially preserving mitochondrial function and maintaining plasma membrane stability. In addition, this study has identified and localised GSTM3 in the tail and equatorial subdomain of the head of boar sperm. Finally, this study has set grounds for future investigations testing supplementation of semen extenders with GSTs, as this may improve fertility outcomes of swine AIs.

## 1. Introduction

Pig breeding worldwide is fundamentally based on the use of artificial insemination (AI). Such a technique is commonly performed through the use of liquid-preserved semen diluted with a proper extender and stored at 15–20 °C, usually for 1 to 5 days [1]; in some cases, however, media may preserve sperm up to 12–15 days [2]. Extender composition and low temperatures contribute to the decrease of sperm metabolic activity, thus maintaining their function and fertilising ability [2]. However, AI-fertility rates using liquid-stored sperm are known to decline within 72 h of its storage [3]. A wide range of changes occurs during liquid-storage of boar semen, including a decrease in sperm motility, viability, and plasma membrane stability as well as an increase of oxidative stress (OS), lipid peroxidation, and apoptotic-like events [3,4].

Reactive oxygen species (ROS) include superoxide anion, hydrogen peroxide, hydroxyl radical, amongst others, and are naturally produced by the activity of the mitochondrial electron transport chain. Depending on their concentration, localisation, or exposure time, the effects of ROS may be both beneficial than harmful [5]. Moreover, OS reflects the imbalance between ROS production and antioxidant sperm capacity [6]. One of the major causes of the decreased sperm quality in liquid-preserved boar semen is the OS-related damage [7]. In comparison to somatic cells, mammalian sperm are highly sensitive to OS due to the high amount of polyunsaturated fatty acids in their plasma membrane phospholipids and their relatively low antioxidant capacity [8]. Along these lines, any factor that initiates OS in sperm, such as low levels of antioxidant protection or high levels of ROS, leads to the induction of upraised OS levels and cell damage as a result of a self-perpetuating redox cycle [9,10]. It has been demonstrated that many antioxidant systems, such as glutathione peroxidases (GPX), glutathione reductases (GSR), catalase (CAT), superoxide dismutase (SOD), and peroxinreductases (PRDX), are capable of regulating physiological levels of ROS in sperm, protecting them from OS (reviewed in [11]). However, the specific role of glutathione S-transferases (GSTs) on mammalian sperm physiology has not been investigated.

GSTs are postulated as important detoxifying enzymes that catalyse reduced glutathione-dependent reactions involved in cellular protection against OS and toxic chemicals [12]. The triple role of sperm GSTs is known to be (i) cell detoxification, which prevents lipid membrane peroxidation and subsequent OS; (ii) cellular signalling regulation involved in sperm capacitation; and (iii) fertilising ability, since GSTM3 is involved in sperm-zona pellucida binding events (reviewed in [13]). Experiments performed in goats evidenced that GSTMs are attached to the sperm plasma membrane by non-covalent interactions [14] and maintain their motility, viability, mitochondrial status, and fertilising ability by preventing lipid peroxidation, and OS [15]. Furthermore, GSTM3 has been recently established as a fertility [16] and cryotolerance [17] biomarker in boar sperm. Related to this, while GSTM3 seems to be the main GST family member in sperm and could play a crucial role in sperm physiology and the maintenance of their function and quality during liquid-storage, its role in sperm cells is yet to be investigated.

Ethacrynic acid (EA) is a well-known inhibitor of GSTs enzymatic activity that strongly and specifically inhibits GSTAs, GSTMs, and GSTPs by blocking their substrate-binding site [18,19]. This blocking effect can occur by direct binding of EA to GSTs as a non-substrate ligand [20] or by conjugation of EA to the thiol group of GSH (EA-GSH), which can be formed either spontaneously or through a GST-catalysed reaction [21].

Along these lines, mounting evidence suggests the essential role of GSTs, and specifically GSTM3, in sperm physiology of goat and boar [13,16,22]. Although OS is known to induce detrimental effects on boar sperm quality parameters during liquid-storage (e.g., impaired motility, viability, and fertilising capacity), the effects of GSTs during semen storage are yet to be investigated in any species. In this regard, understanding the effects and mechanisms of sperm GSTs in liquid-storage of boar semen is of utmost importance, since it may allow improving their preservation and fertilising ability. With this purpose, inhibition of sperm GSTs activity by EA was performed in order to evaluate the effects of GSTs in liquid-preserved boar sperm. Sperm quality and functionality parameters (including motility, mitochondrial membrane potential [i.e., ΔΨm], viability, membrane lipid disorder, acrosome membrane integrity, apoptotic-like changes, intracellular calcium [Ca^2+^] levels and superoxide [i.e., O_2_^−^●], and peroxide [i.e., H_2_O_2_)] levels) were assessed at 0, 24, 48, and 72 h of storage at 17 °C. Additionally, the presence, localisation, and relative levels of GSTM3 in boar sperm were assessed in each treatment after 0 and 72 h of storage at 17 °C.

## 2. Materials and Methods 

### 2.1. Reagents

Ethacrynic acid (EA; Sigma-Aldrich, Saint Louis, MO, USA) was reconstituted in dimethyl sulfoxide (DMSO) to a stock solution of 64 mM. Fluorochromes used for flow cytometry analysis were purchased from Life Technologies (ThermoFisher; Carlsbad, CA, USA) and reconstituted in DMSO, except for propidium iodide (PI) and peanut agglutinin conjugated with fluorescein isothiocyanate (PNA-FITC), which were diluted in phosphate-buffered saline 1× (PBS). The antibody against GSTM3 (ref. ARP53561_P050), as well as its specific blocking peptide (ref. AAP53561), were purchased from Aviva Systems Biology (San Diego, CA, USA). Secondary antibodies for immunoblotting analysis were goat anti-rabbit and rabbit anti-mouse conjugated with horseradish peroxidase (ref. P0448 and ref. P0260; Dako, Derkman A/S; Denmark, respectively), whereas that for immunofluorescence analysis was an anti-rabbit antibody conjugated with Alexa Fluor 488 (ref. A32731; ThermoFisher). 

### 2.2. Animals and Ejaculates

Production of the seminal doses used in this study followed the ISO certification (ISO-9001:2008), and the authors did not manipulate any animal, but they purchased semen doses from a local farm, which operates under commercial, standard conditions. Ejaculates from ten healthy and sexually mature Piétrain boars (n = 10; 1–3 years-old) were provided by an authorised, AI-centre (Grup Gepork S.L., Masies de Roda, Spain). Boars were fed with a standard and balanced diet with water being provided ad libitum, and ejaculates were collected twice a week through the gloved-hand method. Ejaculates were diluted to a final concentration of 30 × 10^6^ spermatozoa/mL with a commercial extender (Androstar^®^ Plus, Minitüb Ibérica, S.L.; Tarragona, Spain), and transported at 17 °C to the laboratory within four hours post-collection. All ejaculates fulfilled the standards of quantity and quality (>200 × 10^6^ spermatozoa/mL, 70% motile spermatozoa, and 75% morphologically normal cells).

### 2.3. Experimental Design

Ten ejaculates (one per boar) were split into two aliquots containing 100 μmol/L of EA and the same volume of DMSO as a vehicle control group. The concentration of DMSO in all treatments was 0.15% (v:v). Inhibitor concentration was selected based on previous studies [23] and preliminary concentration tests performed at our laboratory. All samples were stored for 72 h in closed plastic containers with constantly and gently agitation at 17 °C. Sperm motility and flow cytometry parameters were evaluated at 0, 24, 48, and 72 h, whereas immunofluorescence and immunoblotting analysis against GSTM3 were assessed at 0 and 72 h of semen storage at 17 °C.

### 2.4. Sperm Motility

Prior to sperm motility analysis, 500 µL of each sample was incubated at 37 °C for 10 min. Subsequently, 5 µL of each sperm sample was placed onto a pre-warmed Makler counting chamber (Sefi-Medical Instruments, Haifa, Israel). Motility evaluation was performed through a commercial computer-assisted sperm analysis (CASA) system (Olympus BX41) connected to a computer equipped with ISAS software (Integrated Sperm Analysis System V1.0; Proiser, Valencia, Spain). The following sperm motility parameters were provided by the software: total motility, TMOT (%); progressive motility, PMOT (%); average pathway velocity, VAP (µm/s); curvilinear velocity, VCL (µm/s); straight-line velocity, VSL (µm/s); linearity, LIN (%); beat-cross frequency, BCF (Hz); amplitude of lateral head displacement, ALH (µm) and straightness, STR (%). The sperm motility variables used in the present study were recorded as the percentage of TMOT (average path velocity ≥ 10 μm/sec) and that of motile spermatozoa showing rapid and progressive movement (straightness ≥ 45%). Three replicates per sample, with a minimum of 1000 spermatozoa per replicate, were assessed.

### 2.5. Flow Cytometry Analyses

Sperm plasma membrane integrity (i.e., viability), membrane lipid disorder, acrosome integrity, apoptotic-like changes, ΔΨm, intracellular Ca^2+^ levels, and intracellular levels of O_2_^−^● and H_2_O_2_ were evaluated in each treatment and time. Samples were diluted with pre-warmed PBS to a final concentration of 2 × 10^6^ sperm/mL in a final volume of 0.8 mL and subsequently stained. Flow cytometric analysis was conducted using a Cell Laboratory QuantaSC cytometer (Beckman Coulter; Fullerton, CA, USA) equipped with an argon-ion laser (488 nm) set at a power of 22 mW. Laser voltage and rate were constant throughout the experiment. Unstained and single-stained samples for each fluorochrome were used for setting the electronic volume (EV) gain, FL-1, FL-2, and FL-3 PMT-voltages and for compensating spill over the other channels. EV was used to distinguish the sperm population from debris. Each sample was evaluated three times, with 10,000 events per replicate. Flow cytometric data analysis was performed using Flowing Software (Ver. 2.5.1; University of Turku, Finland), as recommended for the International Society for Advancement of Cytometry.

Sperm viability was evaluated using co-staining with SYBR14 (100 nmol/L) and PI (12 μmol/L) [24]. Membrane lipid disorder of sperm was assessed by merocyanine 540 (M540; 2.6 μmol/L) and YO-PRO-1 (25 nmol/L) co-staining, following the procedure described by Yeste et al. 2014 [25]. Acrosome membrane integrity was assessed by PNA-FITC (2.5 µg/mL), and PI (12 μmol/L) co-staining according to the modified procedure described by Nagy et al. [26]. Apoptotic-like changes in sperm were evaluated by Annexin V and PI co-staining, following the recommended procedure from the Annexin-V-FLUOS Staining Kit (11858777001; Roche Diagnostics, Germany). Levels of ΔΨm were evaluated through 5,5’,6,6’-tetrachloro-1,1’,3,3’tetraethyl-benzimidazolylcarbocyanine iodide (JC1; 0.3 μmol/L) staining [27]. Sperm head and mid-piece Ca^2+^ levels were evaluated through the staining with Fluo3-AM (1 μmol/L) and PI (12 μmol/L) [28,29]. On the other hand, Rhod5-AM (5 μmol/L) and YO-PRO-1 (25 nmol/L) co-staining was performed in order to evaluate head Ca^2+^ deposits exclusively [29,30,31]. Finally, sperm OS was evaluated by assessing intracellular levels of O_2_^−^● and H_2_O_2_, through staining with hydroethidine (HE; 4 μmol/L) and YO-PRO-1 (25 nmol/L) [32] and 2’,7′-dichlorofluorescin diacetate (H_2_DCFDA; 10 μmol/L) and PI (12 μmol/L) [32], respectively. All flow cytometry protocols are described in detail in the Additional File 1 in Supplementary Materials.

### 2.6. Immunofluorescence

Localisation of GSTM3 in boar sperm during liquid preservation was evaluated through immunofluorescence at 0 and 72 h of storage at 17 °C in each treatment. Samples containing 3 × 10^6^ sperm/mL were fixed with 2% (w:v) paraformaldehyde and subsequently washed. The different slides containing two drops per sample were blocked and permeabilised with a blocking solution containing 0.25% (v:v) Triton X-100 and 3% (w:v) Bovine serum albumin (BSA) for 40 min. Then, samples were incubated with a primary anti-GSTM3 antibody (1:250; v:v) overnight. Following this, slides were washed and incubated with an anti-rabbit antibody (1:500; v:v). Then, 10 µL of Vectashield mounting medium containing 4′,6-Diamidino-2-phenylindole dihydrochloride (DAPI) was added. Finally, a coverslip was placed, and samples were sealed with nail varnish. In negative controls, the primary antibody was omitted. For the peptide competition assay, samples were incubated with GSTM3-specific blocking peptide, which was 20 times in excess with regard to the corresponding primary antibody. A confocal laser-scanning microscope (CLSM, Nikon A1R; Nikon Corp., Tokyo, Japan) was used to evaluate all samples.

### 2.7. Immunoblotting

Boar sperm samples of all treatments at 0 and 72 h of storage at 17 °C were used for Western blot analysis. In brief, samples were centrifuged twice at 3000× *g* for 5 min and resuspended in lysis buffer (RIPA Buffer, Sigma-Aldrich) prior to incubation in agitation at 4 °C for 30 min. Triple sonication per sample was carried out, followed by centrifugation at 10,000× *g*, and the supernatant was stored at −80 °C. A detergent compatible method (BioRad; Hercules, CA, USA) was used to quantify total protein. Ten micrograms of total protein were diluted 1:1 (v:v) in Laemmli reducing buffer 2× and boiled at 96 °C for 5 min before proteins were loaded onto the gel and subsequently transferred onto polyvinylidene difluoride (PVDF) membranes using Trans-Blot^®^ Turbo™ (BioRad) and blocked with 5% BSA. Blocked membranes were then incubated with the anti-GSTM3 primary antibody (1:20,000; v:v) overnight. Next, membranes were washed thrice and incubated with the secondary antibody for an hour with agitation (1:35,000; v:v). Finally, bands were visualised using a chemiluminescent substrate (Immobilion^TM^ Western Detection Reagents, Millipore) and scanned with G:BOX Chemi XL 1.4 (SynGene, Frederick, MT, USA). Next, membranes were stripped and blocked prior to incubation with an anti-α-tubulin antibody (1:100,000, v:v) overnight. Subsequently, membranes were washed trice and incubated with an anti-mouse antibody (1:200,000, v:v) for 1 h. Finally, membranes were washed, visualised, and scanned as described previously. The specificity of the primary antibody was confirmed through peptide competition assays utilising GSTM3-immunising peptides, 20 times in excess with regard to the antibody. Bands of three technical replicates per samples were quantified using Quantity One software package (Version 4.6.2; BioRad), and pattern quantifications were normalized using α-tubulin.

### 2.8. Statistical Analysis

Results were analysed using a statistical package (IBM SPSS for Windows 25.0; Armonk, NY, USA). First, data were checked for normality and homogeneity of variances using Shapiro–Wilk and Levene tests, respectively. When required, data were transformed with arcsin √x and then re-assessed for normality and homogeneity of variances. Each statistical case consisted of a separate biological replicate.

Sperm quality and functionality parameters, as well as the relative content of GSTM3, were compared between treatments (EA-treated and control spermatozoa) and throughout storage time (0, 24, 48 and 72 h) with a linear mixed model (repeated measures); within-subjects factor was the time of storage, between-subjects factor was the treatment, and the random-effects factor was the boar. The post-hoc Sidak test was used for pair-wise comparisons. Finally, Pearson correlation coefficients were calculated between the relative content of the GSTM3 band and quality and functionality parameters. Data are shown as mean ± SEM. For all analyses, the level of significance was set at *p* ≤ 0.05.

## 3. Results

All sperm quality and functionality parameters (total and progressive motility, ΔΨm, viability, membrane lipid disorder, acrosome membrane integrity, apoptotic-like changes, intracellular Ca^2+^ levels, and total intracellular O_2_^−^● and H_2_O_2_ levels) of semen samples incubated with EA and the control group were assessed at 0, 24, 48 and 72 h of storage at 17 °C. No differences between groups were found in any sperm quality and functionality parameter at 0 h of storage at 17 °C.

### 3.1. Inhibition of GSTs Impairs Sperm Motility and ΔΨm

**Figure 1:**
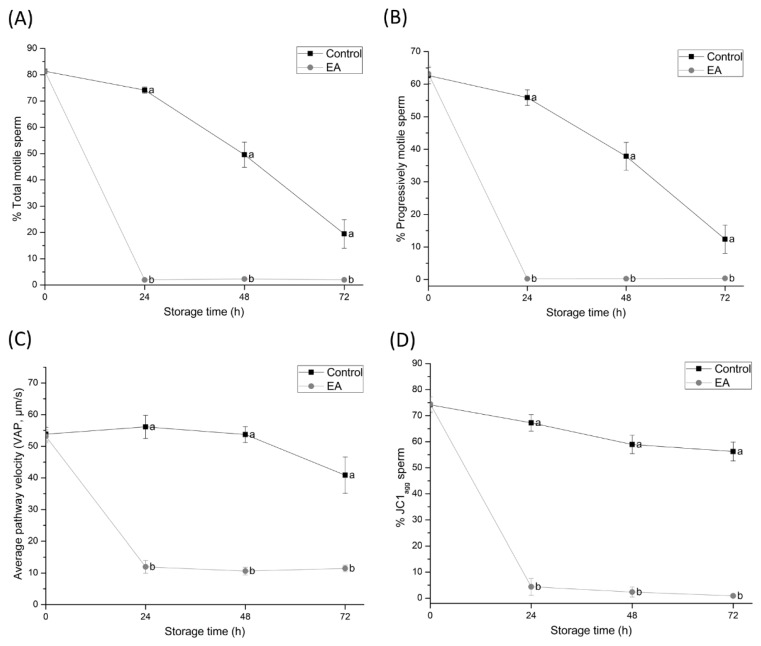
(**A**) percentages of total motile sperm, (**B**) percentages of progressive motile sperm, (**C**) average pathway velocity (VAP; μm/s), and (**D**) percentages of high ΔΨm sperm (JC1_agg_ sperm) of semen samples treated with ethacrynic acid (EA), a glutathione S-transferases (GSTs) inhibitor, and the control group, assessed at different evaluation times during liquid storage at 17 °C (0, 24, 48, and 72 h). Different letters (a, b) indicate significant differences (*p* < 0.05) between treatments within storage time.

Motility was assessed by the percentage of total and progressively motile sperm and the VAP at 0, 24, 48, and 72 h of liquid-storage at 17 °C, whereas sperm mitochondrial function was assessed by the percentage of high ΔΨm resulting from the orange-stained populations (JC1_agg_) (Figure 1).

Compared to the control group, total and progressive motilities and the VAP of EA-treated sperm samples dramatically decreased within the first 24 h of liquid-storage and remained low until 72 h of storage (*p* < 0.05). On the other hand, a dramatic decrease in the percentage of sperm showing high ΔΨm was observed in EA-treated samples compared to the control within the first 24 h of liquid-storage (*p* < 0.05). Moreover, a strong correlation between total motility and ΔΨm was observed (r = 0.873; *p* < 0.01). 

### 3.2. Inhibition of GSTs Causes Sperm Plasma Membrane but not Acrosome Damage

**Figure 2:**
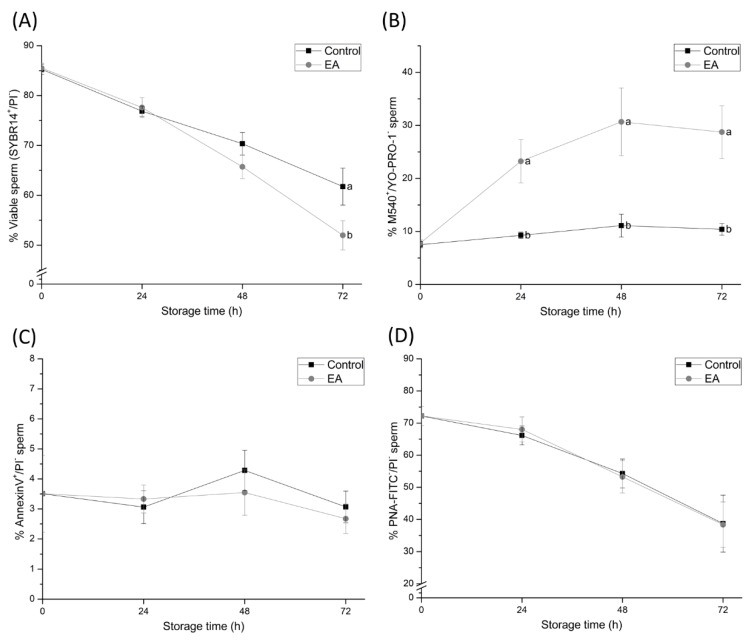
Percentages of (**A**) total viable sperm (SYBR14^+^/PI^-^), (**B**) viable sperm with high membrane lipid disorder (M540^+^/YO-PRO-1^-^), (**C**) viable apoptotic-like sperm (AnnexinV^+^/PI^-^) and (**D**) viable acrosome membrane-intact sperm (PNA-FITC^-^/PI^-^) of semen samples treated with ethacrynic acid (EA), a glutathione S-transferases (GSTs) inhibitor, and the control group, assessed at different evaluation times during liquid storage at 17 °C (0, 24, 48, and 72 h). Different letters (a, b) indicate significant differences (*p* < 0.05) between treatments within storage time.

Sperm plasma membrane status was characterised through SYBR14/PI, M540/YO-PRO-1, PNA-FITC/PI, and Annexin V/PI staining (Figure 2). Although no statistically significant differences in the percentage of viable spermatozoa (SYBR14^+^/PI^-^) were found between control and EA-treated samples at 0, 24, and 48 h of semen storage, a reduced viability was evidenced at 72 h (*p* < 0.05). 

On the other hand, the percentage of sperm with high membrane lipid disorder (M540^+^/YO-PRO-1^-^) was higher in EA-treated samples at 24, 48, and 72 h of liquid-storage (*p* < 0.05). Related to this, the percentage of viable membrane-intact sperm (PNA-FITC^-^/PI^-^) was used to assess acrosome membrane intactness, whereas the percentage of viable Annexin V-positive sperm (Annexin V^+^/PI^-^) was used to assess apoptotic-like changes. EA-treated samples did not show either acrosome membrane damage or apoptotic-like changes at any time-point in comparison to the control group.

### 3.3. Sperm GSTs Are Involved in Ca^2+^ Homeostasis

**Figure 3:**
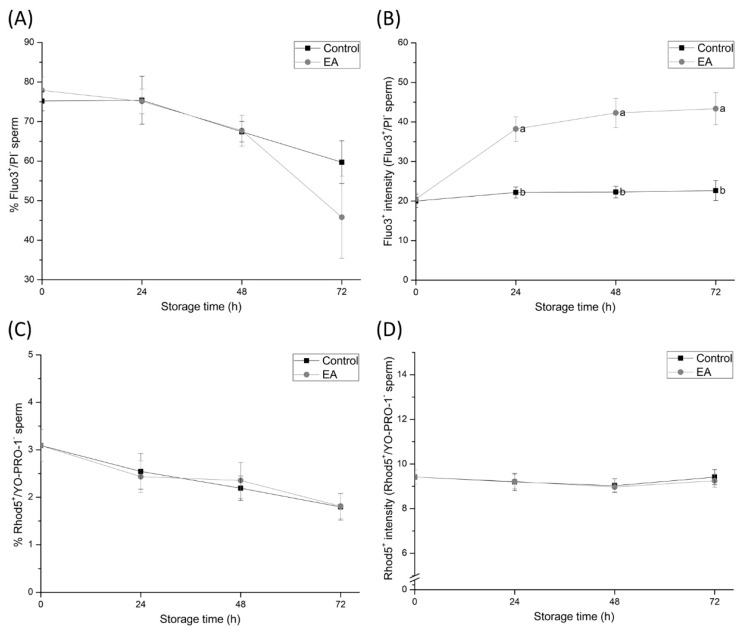
(**A**) Percentages of viable spermatozoa showing high intracellular calcium levels in the mid-piece and head (Fluo3^+^/PI^−^), (**B**) mean Fluo3^+^ fluorescence intensity of viable spermatozoa showing high intracellular calcium levels in the mid-piece and head, (**C**) percentages of viable spermatozoa showing high intracellular calcium levels in the head (Rhod5^+^/YO-PRO-1^–^), and (**D**) mean Rhod5^+^ fluorescence intensity of viable spermatozoa showing high intracellular calcium levels in the sperm head of semen samples treated with ethacrynic acid (EA), a glutathione S-transferases (GSTs) inhibitor, and the control group, assessed at different evaluation times during liquid storage at 17 °C (0, 24, 48, and 72 h). Different letters (a, b) indicate significant differences (*p* < 0.05) between treatments within storage time.

The percentage and fluorescence intensity of viable spermatozoa showing high Ca^2+^ levels (Fluo3^+^/PI^-^ and Rhod5^+^/YO-PRO-1^-^) were used to assess sperm intracellular Ca^2+^ levels (Figure 3). Although no differences in the percentage of Fluo3^+^/PI^-^ sperm were found, Fluo3^+^/PI^-^ fluorescence intensity in EA-treated spermatozoa was higher in comparison to the control group after 24, 48 and 72 h of liquid preservation (*p* < 0.05), showing increased Ca^2+^ levels in GSTs-inhibited samples. On the other hand, percentages of Rhod5^+^/YO-PRO-1^-^ sperm and Rhod5^+^-fluorescence intensity did not show differences between treatments at any time-point of semen storage at 17 °C.

### 3.4. Sperm GSTs are Involved in Intracellular ROS Regulation

**Figure 4:**
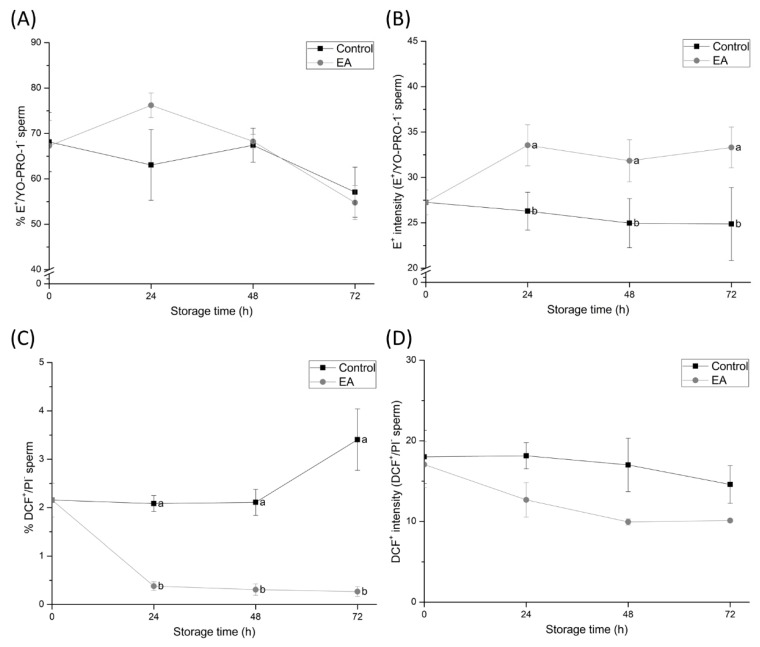
(**A**) Percentages of viable spermatozoa showing high superoxide (O_2_^-^•) levels (E^+^/YO-PRO-1^−^), (**B**) mean E^+^ fluorescence intensity of viable spermatozoa showing high superoxide levels, (**C**) percentages of viable spermatozoa showing high peroxide (H_2_O_2_) levels (DCF^+^/PI^−^), and (**D**) mean DCF^+^ fluorescence intensity of viable spermatozoa showing high peroxide levels of semen samples treated with ethacrynic acid (EA), a glutathione S-transferases (GSTs) inhibitor, and the control group, assessed at different evaluation times during liquid storage at 17 °C (0, 24, 48, and 72 h). Different letters (a, b) indicate significant differences (*p* < 0.05) between treatments within storage time.

Percentages of E^+^/YO-PRO-1^-^ and DCF^+^/PI^−^ sperm and fluorescence intensities of E^+^ and DCF^+^ were assessed to evaluate intracellular levels of O_2_^−^● and H_2_O_2_ (Figure 4). An increase of O_2_^−^● in sperm cells due to GSTs inhibition was detected, since E^+^-fluorescence intensity, although not the percentage of E^+^/YO-PRO-1^-^ sperm, in EA-treated samples was higher than the control at 24, 48, and 72 h of liquid-storage (*p* < 0.05). On the other hand, a decrease in the percentage of H_2_O_2_-positive sperm cells (DCF^+^/PI^−^ sperm) was found due to GSTs inhibition at any evaluation time (*p* < 0.05), even though the mean fluorescence intensity of DCF^+^ did not differ from the control group.

### 3.5. GSTM3 Partially Disappear from the Boar Sperm Mid-Piece during Liquid-Storage

**Figure 5:**
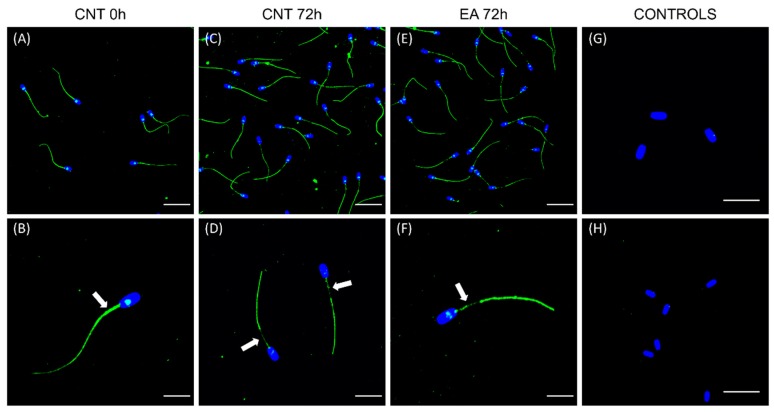
Immunolocalisation of sperm GSTM3. (**A**,**B**) control group at 0 h of storage at 17 °C, (**C**,**D**) control group at 72 h of liquid-storage, (**E**,**F**) Ethacrynic acid (EA)-treated spermatozoa at 72 h of liquid-storage, (**G**) negative control, and (**H**) peptide competition assay. White arrows indicate the sperm midpiece. The nucleus is shown in blue colour (DAPI), whereas GSTM3 is shown in green (fluorescein isothiocyanate, FITC). Scale bars: A, C, E, H: 30 μm; D, G: 15 μm; B, F: 10 μm.

Localisation of GSTM3 was resolved at 0 and 72 h of storage at 17 °C by immunofluorescence. Figure 5 shows representative localisation patterns of GSTM3 in GSTs-inhibited and non-inhibited sperm samples at 0 and 72 h of liquid-storage at 17 °C. All sperm cells showed the GSTM3 fluorescence signal, and the negative control and peptide competition assay confirmed the specificity of the GSTM3 antibody. GSTM3 was found to be localized in the mid, principal, and end pieces of the tail and the equatorial subdomain of the head in sperm samples at 0 h of liquid-storage. However, after 72 h of semen storage at 17 °C, the GSTM3 signal partially disappeared from the mid-piece in both the control and GSTs-inhibited samples.

### 3.6. Sperm GSTM3 Content Was Reduced During Sperm Liquid Preservation

**Figure 6:**
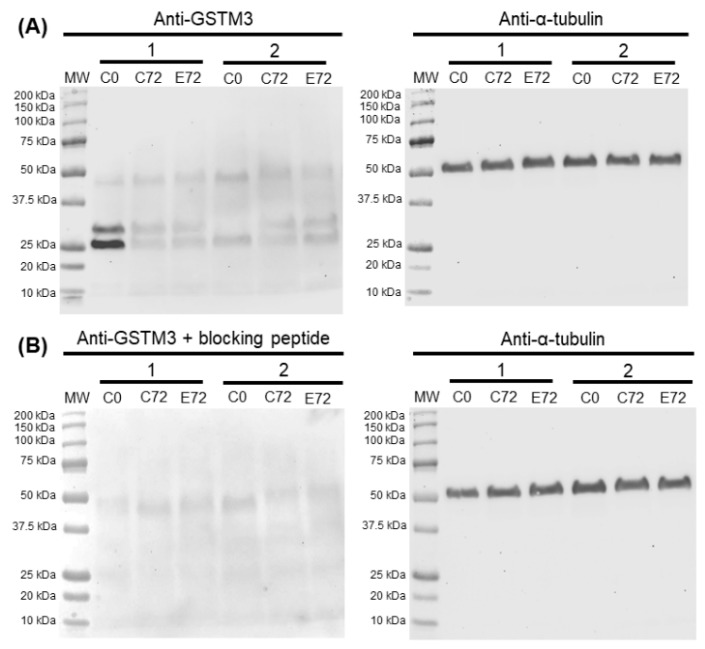
(**A**) Representative Western blots resulting from the incubation with the GSTM3 antibody (Anti-GSTM3) and its loading control (α-tubulin). (**B**) Western blots resulting from incubation with the GSTM3 antibody with GSTM3-blocking peptide (Anti-GSTM3 + blocking peptide) and its loading control (α-tubulin). Lanes MW: molecular weight. Lanes C0: control at 0 h of sperm liquid storage. Lanes C72: control at 72 h of sperm liquid storage. Lanes E72: EA-treated samples at 72 h of liquid storage.

Immunoblotting analysis of GSTM3 showed a triple-band pattern of ~25, ~28, and ~48 kDa in every experimental condition. Peptide competition assay utilising GSTM3 immunising peptide confirmed both ~25 (GSTM3-A) and ~28 (GSTM3-B) kDa-bands as GSTM3-specific (Figure 6).

**Figure 7:**
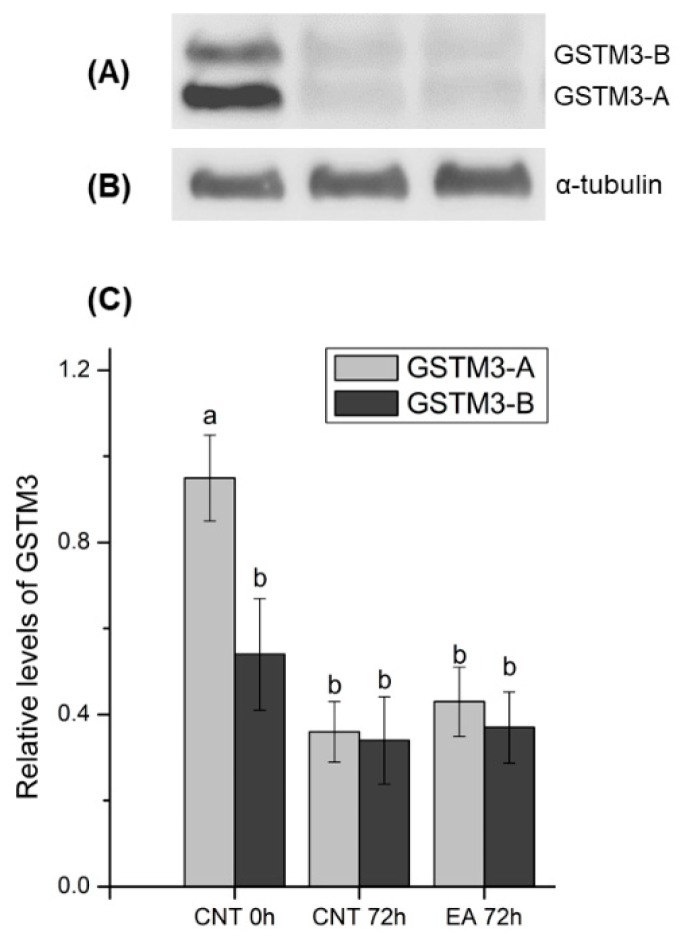
(**A**) Representative Western blot resulting from incubation with the GSTM3 antibody and (**B**) its loading control (α-tubulin). (**C**) Relative abundances of ~25 (GSTM3-A) and ~28 (GSTM3-B) kDa bands as mean ± standard error of the mean in all treatments. Values were normalised using the α-tubulin protein as an internal standard. Each sperm sample (n = 10) was evaluated two times. CNT 0 h: control at 0 h of sperm liquid storage; CNT 72 h: control at 72 h of sperm liquid storage; EA 72 h: Ethacrynic acid (EA)-treated samples at 72 h of liquid storage. Different letters (a, b) indicate significant differences (*p* < 0.05) between treatments.

As shown in Figure 7, normalised GSTM3-A content was found to be significantly higher than GSTM3-B after 0 h of storage at 17 °C (*p* < 0.05). Additionally, at 0 h, GSTM3-A was significantly higher than GSTM3-A and GSTM3-B after 72 h of storage in control and GSTs-inhibited groups (*p* < 0.05). However, the relative abundance of GSTM3 in GSTs-inhibited samples at 72 h of liquid storage did not differ from the control.

### 3.7. Relative Content of GSTM3 Was Highly Correlated with ΔΨm and Motility

Pearson correlation coefficients of relative content of GSTM3-A and GSTM3-B at 0 h with sperm quality and functionality parameters of liquid-stored sperm at 72 h are shown in Table 1. No correlation between the relative abundance of GSTM3-A relative and any sperm quality or functionality parameters was found. However, the relative abundance of GSTM3-B was negatively correlated with total and progressive sperm motility and ΔΨm (JC1_agg_) (*p* < 0.05).

## 4. Discussion

Preservation of boar semen in liquid storage at 17 °C leads to a decrease in sperm metabolic activity in order to maintain their function and fertilising ability [2]. However, sperm liquid-preservation may result in impaired motility, viability, membrane stability, OS, and apoptotic-like changes [3,4]. GSTs in sperm are membrane-attached, detoxifying enzymes [14], which have been considered to be fertility [16], and cryotolerance [17] biomarkers in boar sperm. Furthermore, previous studies have shown that extender supplementation with glutathione decreases OS and improves the quality of boar semen during liquid storage at 17 °C [33]. Such findings suggest that GSTs play a vital role in maintaining sperm physiology during liquid preservation. However, its effects upon quality and functionality parameters of sperm have never been investigated. Findings from this work are in accordance with the aforementioned studies since GSTs-inhibition during boar semen storage was found to decrease sperm quality and function parameters dramatically. 

The most noticeable effect of GSTs-inhibition was evidenced by the complete loss of total and progressive motility and a significant reduction in VAP within the first 24 h of semen liquid-storage at 17 °C. Such motility impairment is in agreement with previous studies performed in the goat, where sperm motility is known to decrease due to GSTs-inhibition [23]. Furthermore, the fact that GSTM3 was localised along the principal piece of boar sperm supports these results. In addition, not only did JC1 staining show a dramatic decrease in ΔΨm due to GSTs-inhibition, which was also described in goat sperm by Hemachand and Shaha [34], but ΔΨm was strongly and positively correlated with total motility. Correlation between these factors has been extensively reported in the literature, as adenosine triphosphate (ATP) production [35] and controlled ROS levels [36] are known to be required for proper sperm motility. Together, these findings suggest that sperm GSTs play an essential role in regulating mitochondrial function and motility performance during liquid-storage of boar semen.

The results of the present study have also confirmed that sperm plasma membrane status is impaired by GSTs-inhibition. Although the percentage of viable sperm was not significantly affected until 72 h of semen liquid-storage at 17 °C, the percentage of viable sperm with high membrane lipid disorder dramatically increased within the first 24 h of semen storage. Such findings are in agreement with previously-reported studies confirming that GSTs function is mainly located in the sperm plasma membrane [34], and their inhibition causes sperm membrane damage in goat sperm [23]. Along these lines, the present study provides evidence confirming that membrane-bound GSTs prevent cholesterol efflux and membrane lipid disorder, and thus delay capacitation-like changes in liquid-stored boar sperm. However, further experiments regarding the specific role of GSTs in sperm capacitation should be performed in order to clarify their specific role in the changes in the sperm plasma membrane. 

Although the inhibition of sperm GSTs during liquid-storage was found to cause sperm membrane destabilisation, the acrosome membrane remained intact. These findings suggest that despite GSTs-inhibition increasing the lipid disorder of the sperm plasma membrane and causing capacitation like-changes in sperm cells, GSTs do not exert a direct effect on the acrosome membrane. On the other hand, and in agreement with the maintenance of sperm viability during the first hours of semen storage, apoptotic-like changes in sperm do not increase due to GSTs inhibition. Such results suggest that GSTs are not involved in apoptotic-like processes in sperm during boar semen liquid-storage. Likewise, GSTs were found to be involved in sperm intracellular Ca^2+^ content release. Intracellular Ca^2+^ levels from the mid-piece and sperm head were observed to increase within 24 h of semen storage due to GSTs-inhibition, whereas Ca^2+^ levels in sperm head did not. These findings indicate that the inhibition of sperm GSTs augment Ca^2+^ levels in the sperm mid-piece rather than in the head. While mitochondrial Ca^2+^ signalling is not completely understood, such organelles are known to function as intracellular Ca^2+^ stores, since the negatively charged mitochondrial matrix can sequester Ca^2+^ ions [36]. The impairment of mitochondrial Ca^2+^ homeostasis due to GSTs-inhibition may be caused by the destabilisation of mitochondrial membranes. However, further research is required in order to elucidate this hypothesis. In spite of the aforementioned, these results evidence, altogether, the crucial role of sperm GSTs in the regulation of mitochondrial Ca^2+^ homeostasis during liquid-storage of boar semen.

Our results also demonstrated that the inhibition of GSTs led to changes in the physiological ROS levels of sperm during storage at 17 °C. Although the percentage of O_2_^−^•-positive sperm increased because of GSTs-inhibition, intracellular levels of H_2_O_2_ decreased. The fact that the main ROS source in sperm is thought to reside in the mitochondria [37], which have been shown to be impaired by GSTs-inhibition, supports the apparent role of GSTs in sperm ROS production. Impaired mitochondrial activity by GSTs-inhibition may contribute to the formation, but not removal of the O_2_^−^● in sperm [38], which could explain the high percentage of O_2_^−^●-positive sperm in GSTs-inhibited samples. Interestingly, while H_2_O_2_ is generated by SOD using O_2_^−^● as substrate, H_2_O_2_ levels in GSTs-inhibited sperm were seen to decrease. This apparent contradiction can be easily addressed. In addition to GSTs, other relevant antioxidant systems in sperm, such as GPX, CAT, and PRDX, have been shown to modulate physiological H_2_O_2_ levels [39]. Furthermore, the formation of H_2_O_2_ by SOD could be reduced due to GSTs inhibition, since this NADPH-dependent enzyme is blocked by the lack of reducing power caused by mitochondrial impairment. Consequently, the arrest in H_2_O_2_ generation but the continuous removal of this electrophilic compound could explain its reduction during GSTs-inhibition. However, the analysis of NADPH generation would be required in order to confirm this hypothesis. Along these lines, the inhibition of the detoxification function in sperm GSTs was found to enhance the formation of O_2_^−^● and to reduce that of H_2_O_2_. These results unveil the essential role played by GSTs, which, together with other antioxidant systems, regulate physiological ROS levels in sperm and protect them from OS [11]. Therefore, our study could serve as a basis for further studies aimed at clarifying the specific role of GSTs during sperm capacitation and fertilisation, as physiological ROS levels are essential for both processes.

Results from the immunoblotting analysis of GSTM3 showed a specific two-band pattern consisting of ~ 25 (GSTM3-A) and ~ 28 (GSTM3-B) kDa-bands, and a non-specific band of ~48 kDa. The double-band pattern found in the present work could be caused as a result of post-translational modifications of GSTM3 such as phosphorylation, acetylation or glycosylation, among others, which are widely reported in the literature (reviewed by [40]). Quantification of both bands showed higher relative levels of GSTM3-A than GSTM3-B at 0 h of liquid storage. Furthermore, GSTM3-A at 0 h showed higher relative levels than GSTM3-A and GSTM3-B after 72 h of liquid storage in control and GSTs-inhibited samples. Therefore, the results shown herein indicate that a loss of GSTM3 content occurs during liquid storage at 17 °C. However, inhibition of GSTs does not induce changes in the GSTM3 content. In this regard, the preservation of boar semen in liquid storage could induce GSTM3 loss and, consequently, impairment of its function. Nevertheless, a specific assay confirming the presence of post-translational modifications should be performed to gain further insights into the molecular action of GSTM3 in sperm.

The localisation patterns of GSTs in boar sperm during liquid preservation has been established for the first time in the present study. Sperm GSTM3 was localised in the mid, principal, and end pieces of the tail and the equatorial subdomain of the head of samples at 0 h of liquid-storage. This localisation pattern is similar to that found in the boar [17] and other species, such as the buffalo [41]. Moreover, the localisation of GSTM3 in the sperm tail would contribute to explaining the dramatic effect of GSTs-inhibition upon sperm motility and mitochondrial function. Interestingly, the GSTM3 signal was found to be partially reduced from the mid-piece during boar semen liquid-storage in both the control and GSTs-inhibited samples. Since immunoblotting analysis found GSTM3 content to be reduced during semen storage, it becomes apparent that such enzyme is lost rather than relocalised from the mid-piece during liquid-storage. Contrary to the results of the present study, GSTM3 was reported to relocalise to the mid-piece following boar sperm cryopreservation [17].

Finally, the present study also attempted to find a relationship between sperm quality and functionality parameters after 72 h of liquid storage and the relative amounts of GSTM3 at 0 h. Interestingly, a negative correlation between relative levels of GSTM3-B at 0 h and motility and mitochondrial function after 72 h of sperm preservation was observed. Mounting evidence in the literature supports the relationship between GSTM3 and mitochondrial function, since GSTM3 content is known to be higher in mitochondrial-altered sperm of men [42]. Moreover, recent studies demonstrated that GSTM3 content in fresh sperm is highly correlated to the mitochondrial activity of frozen-thawed sperm, and relocalisation of this enzyme from the entire tail to the mid-piece occurs during cryopreservation of boar [17] and buffalo [41] sperm. Therefore, the relationship between GSTM3 content and mitochondrial activity found in the present study strengthens the hypothesis of a tight molecular relationship between sperm GSTs and mitochondrial function. Moreover, GSTM3 is clearly related to sperm quality, as it has been established as a quality [42,43,44], fertility [16], and cryotolerance [17] biomarker in both boar and human sperm. Hence, one could suggest that the GSTM3 content in fresh boar semen may be used as a biomarker of sperm quality during liquid preservation.

## 5. Conclusions

In conclusion, the data reported in the present study revealed the essential role of membrane-attached sperm GSTs to preserve sperm function and quality in liquid-stored boar semen. Specifically, inhibition of sperm GSTs evidenced that these enzymes are highly related to the preservation of mitochondrial function and maintenance of the plasma membrane stability, thus preserving sperm motility, maintaining physiological ROS levels, and regulating mitochondrial Ca^2+^ homeostasis. In addition, this study identified and localised GSTM3 for the first time in boar sperm during storage at 17 °C for 72 h. GSTM3 was localised in the mid, principal and end pieces of the tail and the equatorial subdomain of the head, and was partially lost from the mid-piece after 72 h of liquid preservation. Matching with this, immunoblotting showed that the relative amounts of sperm GSTM3 decreased after 72 h of liquid storage at 17 °C. Additionally, relative GSTM3-content at 0 h of storage was negatively correlated to sperm mitochondrial function and motility after 72 h of storage, supporting the mitochondrial-protective role of GSTs and suggesting GSTM3 as a putative biomarker of sperm quality during semen liquid-storage. Finally, while the molecular role of GSTs on sperm physiology and specifically on mitochondrial function is yet to be elucidated, the findings reported in this study warrant further research testing the supplementation of boar semen extender with GSTs, as this may preserve sperm mitochondrial function and plasma membrane stability during liquid storage and improve subsequent reproductive performance of boar AI-doses.

## Figures and Tables

**Table 1:** Pearson correlation coefficients between the relative GSTM3-A and GSTM3-B abundance at 0 h and sperm quality and functionality parameters at 72 h of liquid storage at 17 °C.

Sperm Quality and Functionality Parameters.	GSTM3-A	GSTM3-B
% total motile sperm	0.27	−0.93 **
% progressively motile sperm	0.18	−0.92 **
% high mitochondrial membrane potential sperm (JC1_agg_)	0.03	−0.87 *
% total viable sperm (SYBR14^+^/PI^-^)	−0.15	0.45
% viable lipid membrane-destabilised sperm (M540^+^/YO-PRO-1^-^)	0.29	−0.48
% viable membrane-intact sperm (PNA-FITC^-^/PI^-^)	−0.05	0.10
% viable apoptotic-like spermatozoa (Annexin V-FITC^+^/PI^-^)	−0.05	0.34
% viable high-Ca^2+^ sperm (Fluo3^+^/PI^-^)	−0.66	0.88
% viable high-Ca^2+^ sperm (Rhod5^+^/YO-PRO-1^-^)	0.29	0.86
% viable high- H_2_O_2_ sperm (DCF^++^/PI^-^)	0.02	−0.70

*p < 0.05; **p < 0.01.

## Supplementary Materials

The following are available online at https://www.mdpi.com/2076-3921/9/2/100/s1, Additional File 1: supplementary information for Materials and Methods.
