# Supplementary material for: Glutathione S-Transferases Play a Crucial Role in Mitochondrial Function, Plasma Membrane Stability and Oxidative Regulation of Mammalian Sperm
Source: Antioxidants (Basel). 2020 Jan 24;9(2):100. doi: 10.3390/antiox9020100 (PMC7070295; doi:10.3390/antiox9020100)
Supplement: Supplementary file 1 [file antioxidants-09-00100-s001.pdf]

Supplementary information for Materials and Methods

Flow cytometry analyses

General information

Plasma membrane integrity [i.e. viability], membrane lipid disorder, mitochondrial membrane potential [i.e. ΔΨm], intracellular levels of superoxides [i.e. O_2^{-}●] and peroxides [i.e. H_2O_2], midpiece and head calcium levels, acrosome membrane integrity and apoptosis-like changes) were evaluated in every treatment and time.

Samples were diluted with pre-warmed PBS to a final concentration of 2×10^6 cells per mL in a final volume of 0.8 mL before they were stained with the corresponding protocol. The flow cytometry assessments were conducted using a Cell Laboratory QuantaSC cytometer (Beckman Coulter; Fullerton, CA, USA), and samples were excited with an argon ion laser (488 nm) set at a power of 22 mW. Laser voltage and rate was constant along the experiment. The cytometer provided the electronic volume (EV) and the side scatter (SS) for each event. Three optical filters (FL-1, FL-2 and FL-3) were used. FL-1 served to detect green fluorescence (SYBR14, YO-PRO-1, 5,5',6,6'-tetrachloro-1,1',3,3'tetraethyl-benzimidazolylcarbocyanine iodide monomers (JC1 monomers; $JC1_{mon}$), peanut agglutinin conjugated with fluorescein isothiocyanate (PNA-FITC), 2',7′-dichlorofluorescin diacetate (H_2DCFDA), Fluo-3-AM and Annexin V-FITC), FL-2 was used to detect orange fluorescence (JC1 aggregates; $JC1_{agg}$) and FL-3 allowed to detect red fluorescence (merocyanine 540 (M540), hydroethidine (HE) and propidium iodide (PI)). Signals were logarithmically amplified, and photomultiplier settings were adjusted to particular staining methods.

Unstained and single-stained samples for each fluorochromes were used for setting the electronic volume (EV) gain, FL-1, FL-2 and FL-3 PMT-voltages and for compensating spill over other channels. Sperm population was distinguished from debris using the EV. A

total of three technical replicates, with 10,000 events per replicate, were evaluated for each sample and sperm parameter. Flowing Software (Ver. 2.5.1; University of Turku, Finland) was used to perform flow cytometric data analysis, following the recommendations of the International Society for Advancement of Cytometry (ISAC). The corresponding mean ± standard error of the mean (SEM) was subsequently calculated.

Plasma membrane integrity (SYBR14/PI)

Sperm viability was evaluated by assessing their membrane integrity using the LIVE/DEAD sperm viability kit (Molecular Probes, Eugene, OR, USA), which follows the protocol of Garner and Johnson [1]. Briefly, spermatozoa were stained with SYBR14 (final concentration: 100 nmol/L) for 10 min at 38ºC in the dark and with PI (final concentration: 12 µmol/L) for 5 min at the same conditions. Combination of SYBR14 and PI resulted in three sperm populations in flow cytometry dot-plots: (i) viable green-stained spermatozoa ($SYBR14^+/PI^-$); (ii) non-viable red-stained spermatozoa ($SYBR14^-/PI^+$); and (iii) moribund spermatozoa stained both green and red ($SYBR14^+/PI^+$). Non-sperm particles (debris; $SYBR14^-/PI^-$) appeared in the left-bottom quadrant. Viable green-stained spermatozoa ($SYBR14^+/PI^-$) were used to assess sperm viability. SYBR14 spill over into the FL3-channel was compensated (2.45%). The percentage of particles within the double negative (left bottom) quadrant of every parameter described above was corrected using the debris particles found in $SYBR14^-/PI^-$ population. The percentages of the other three populations were also recalculated.

Sperm membrane lipid disorder (M540/YO-PRO-1)

Membrane lipid disorder of sperm was evaluated by M540 and YO-PRO-1 co-staining, following the procedure of Rathi et al. [2] with minor modifications by Yeste et al. 2014 [3]. Briefly, spermatozoa were incubated with M540 (final concentration: 2.6 µmol/L) and

YO-PRO-1 (final concentration: 25 nmol/L) for 10 min at 38ºC in the dark. M540 is a hydrophobic fluorochrome which is able to intercalate into the membrane. As membrane fluidity increases M540 uptake, this fluorochrome is stablished a marker for destabilisation of sperm plasma membrane validated in boar, bull, stallion and dog sperm [4]. The combination of both fluorochromes resulted in four populations: (i) viable spermatozoa with low membrane lipid disorder ($M540^-$/YO-PRO-1^-); (ii) viable spermatozoa with high membrane lipid disorder ($M540^+$/YO-PRO-1^-); (iii) non-viable spermatozoa with low membrane lipid disorder ($M540^-$/YO-PRO-1^+); and (iv) non-viable spermatozoa with high membrane lipid disorder ($M540^+$/YO-PRO-1^+). Data were not compensated. Data was not compensated. Viable red-stained spermatozoa ($M540^+$/YO-PRO-1^-) were used to assess membrane lipid disorder.

Mitochondrial membrane potential (JC1)

ΔΨm of sperm was evaluated following a modified protocol from Ortega-Ferrusola et al. [5]. All samples were incubated with JC1 (final concentration: 0.3 µmol/L) for 30 min at 38ºC in the dark. High ΔΨm causes JC1 aggregates that emit orange fluorescence collected through FL-2, whereas low ΔΨm causes JC1 monomers that emit green fluorescence collected through FL-1. Consequently, a total of three sperm populations were observed in flow cytometry dot-plots: (i) spermatozoa with low ΔΨm (green-stained); (ii) spermatozoa with high ΔΨm (orange-stained); and (iii) spermatozoa with heterogeneous mitochondria (green and orange-stained in the same cell). Data was not compensated. Spermatozoa considered having high ΔΨm ($JC1_{agg}$), resulted from the orange-stained populations, were used to assess ΔΨm.

Total intracellular O_2^-● levels (HE/YO-PRO-1)

Total sperm intracellular superoxide (O_2^-●) levels were evaluated following a modification of the procedure described by Guthrie and Welch [6]. Sperm samples were incubated with HE (final concentration of 4 µmol/L) and with YO-PRO-1 (final concentration of 40 nmol/L), at 38°C and for 20 min in the dark. The oxidation of HE to ethidium (E^+) by O_2^-● was detected as red fluorescence through FL-3 and green fluorescence from YO-PRO-1 was detected through FL-1. Combination of these fluorochromes resulted in four populations: (i) non-viable spermatozoa with high O_2^-● levels (E^+/YO-PRO-1^+), (ii) viable spermatozoa with high O_2^-● levels (E^+/YO-PRO-1^-), (iii) non-viable spermatozoa with low O_2^-● levels (E^-/YO-PRO-1^+); and (iv) viable spermatozoa with low O_2^-● levels cells (E^-/YO-PRO-1^-). YO-PRO-1 spill over into the FL3-channel was compensated (5.06%). Spermatozoa considered having high O_2^-● levels (E^+/YO-PRO-1^-) were used to asses O_2^-● levels.

Total intracellular H_2O_2 levels (H_2DCFDA/PI)

Total intracellular peroxides (H_2O_2) levels were evaluated following the modified procedure described by Guthrie & Welch [6]. Samples were incubated with H_2DCFDA (final concentration: 10 µmol/L) for 30 min at 38 ºC in the dark. Subsequently, cells were stained with PI (final concentration: 12 µmol/L) for 10 min at 38ºC. The oxidation of H_2DCFDA to DCF^+ by H_2O_2 was collected through FL-1 as green fluorescence, whereas red fluorescence from PI was detected through FL-3. The following four sperm populations were distinguished in the dot plots: (i) non-viable spermatozoa with high peroxide levels (DCF^+/PI^+); (ii) viable spermatozoa with high peroxide levels (DCF^+/PI^-); (iii) non-viable spermatozoa with low peroxide levels (DCF^-/PI^+); and (iv) viable spermatozoa with low peroxide levels (DCF^-/PI^-). Data was not compensated. Viable spermatozoa considered having high H_2O_2 levels (DCF^+/PI^-) were used to asses H_2O_2 levels.

Acrosome membrane integrity analysis (PNA-FITC/PI)

Sperm acrosome membrane intactness was evaluated according to the modified procedure described by Nagy et al. [7]. Sperm samples were incubated with PNA-FITC (final concentration: 2.5 µg/mL) for 5 min at 38 ºC in the dark. Subsequently, cells were stained with PI (final concentration: 12 µmol/L) for 5 min at 38ºC. PNA-FITC is a hydrophobic enzyme that binds to the outer acrosomal membrane of sperm [8]. As spermatozoa were not previously permeabilised, the four following sperm populations were distinguished: i) viable membrane-intact spermatozoa ($PNA-FITC^-/PI^-$); ii) non-viable spermatozoa with damaged plasma membrane that presented an outer acrosome membrane that could not be fully intact ($PNA-FITC^+/PI^+$); iii) non-viable spermatozoa with damaged plasma membrane and fully-lost outer acrosome membrane ($PNA-FITC^-/PI^+$); and iv) viable spermatozoa with damaged plasma membrane ($PNA-FITC^+/PI^-$). Compensation of PNA-FITC spill over into the PI channel (2.45%) was performed. Viable membrane-intact spermatozoa ($PNA-FITC^-/PI^-$) were used to assess acrosome membrane integrity.

Intracellular calcium levels (Fluo3-AM/PI)

Midpiece and head intracellular calcium of spermatozoa was determined using the modified protocol described by Harrison et al. [9]. Samples were incubated for 10 min at 38ºC with Fluo3-AM (final concentration: 1 µmol/L) and PI (final concentration: 12 µmol/L). Fluo3-AM is a non-fluorescent non-polarized membrane-permeable dye that, upon binding to calcium, emit green fluorescence [10]. Previous studies in boar sperm showed that Fluo3-AM marks both head and midpiece calcium deposits [11]. Accordingly, four sperm populations were identified in dot plots: i) viable spermatozoa with low levels of intracellular calcium ($Fluo3-AM^-/PI^-$); ii) viable spermatozoa with high levels of intracellular calcium ($Fluo3-AM^+/PI^-$); iii) non-viable spermatozoa with low levels of intracellular calcium ($Fluo3-AM^-/PI^+$); and iv) non-viable spermatozoa with high levels of

intracellular calcium (Fluo3-AM$^+$/PI$^+$). Compensation of Fluo3-AM spill over into FL-3 (2.45%) and PI spill over into FL-1 (28.72%) was performed. Viable spermatozoa with high intracellular calcium levels (Fluo3-AM$^+$/PI$^-$) were used to assess sperm head and midpiece calcium levels.

Intracellular calcium levels (Rhod5-AM/YO-PRO-1)

Head intracellular calcium levels were evaluated by Rhod5-AM/YO-PRO-1 co-staining following the modified procedure of Hayato et al., 2011 [12] and De la Fuente et al, 2012 [13], adapted for boar sperm by Yeste et. al., 2015 [11]. Spermatozoa were stained with Rhod-5-AM (final concentration: 5 µmol/L) and YO-PRO-1 (final concentration: 25 µmol/L) and incubated at 38°C for 10 min. Contrary to Fluo3-AM, Rhod5-AM is a polarized membrane-permeable dye with lower calcium-affinity [10], that specifically marks head calcium deposits [11]. A total of four sperm populations appeared in the dot plot: (i) viable spermatozoa with low levels of intracellular calcium (Rhod5-AM$^-$/YO-PRO-1$^-$); (ii) viable spermatozoa with high levels of intracellular calcium (Rhod5-AM$^+$/YO-PRO-1$^-$); (iii) non-viable spermatozoa with low levels of intracellular calcium (Rhod5-AM$^-$/YO-PRO-1$^+$); and (iv) non-viable spermatozoa with high levels of intracellular calcium (Rhod5-AM$^+$/YO-PRO-1$^+$). Fluorescence from YO-PRO-1 over into FL-3 (5.06%) and Rhod5-AM over into FL-1 (3.16%) was compensated. Viable spermatozoa with high levels of intracellular calcium (Rhod5-AM$^+$/YO-PRO-1$^-$) were used to assess the sperm head calcium levels.

Apoptosis-like changes (AnnexinV-FITC/PI)

Apoptosis-like changes of sperm were assessed following the recommended procedure from the Annexin-V-FLUOS Staining Kit (11858777001; Roche Diagnostics, Germany). During apoptosis, phosphatidylserine (PS) is translocated from the inner to the outer side of

plasma membrane, where AnnexinV-FITC binds to it. All samples were incubated with AnnexinV-FITC for 10 min at 38ºC in the dark. Following PI was incubated 10 min in the same conditions. Consequently, a total of four sperm populations were observed in flow cytometry dot-plots: (i) viable non-apoptotic spermatozoa (AnnexinV-FITC$^-$/PI$^-$); (ii) spermatozoa exhibiting apoptotic-like changes (AnnexinV-FITC$^+$/PI$^-$); (iii) non-viable spermatozoa without PS exposed on the outer membrane (AnnexinV-FITC$^-$/PI$^+$); and (iv) necrotic spermatozoa (AnnexinV-FITC$^+$/PI$^+$). FITC spill over into the FL3-channel was compensated (2.45%). Apoptotic-like spermatozoa (AnnexinV-FITC$^+$/PI$^-$) were used to assess apoptosis-like changes.

References cited in Supplementary Information for Materials and Methods

1. Garner, D.L.; Johnson, L.A. Viability assessment of mammalian sperm using SYBR-14 and propidium iodide. *Biol. Reprod.* **1995**, *53*, 276–84.
2. Rathi, R.; Colenbrander, B.; Bevers, M.M.; Gadella, B.M. Evaluation of in vitro capacitation of stallion spermatozoa. *Biol. Reprod.* **2001**, *65*, 462–70.
3. Yeste, M.; Estrada, E.; Rivera del Álamo, M.-M.; Bonet, S.; Rigau, T.; Rodríguez-Gil, J.-E. The increase in phosphorylation levels of serine residues of Ppotein HSP70 during holding time at 17°C is concomitant with a higher cryotolerance of boar spermatozoa. *PLoS One* **2014**, *9*, e90887.
4. Steckler, D.; Stout, T.A.E.; Durandt, C.; Nöthling, J.O. Validation of merocyanine 540 staining as a technique for assessing capacitation-related membrane destabilization of fresh dog sperm. *Theriogenology* **2015**, *83*, 1451–1460.
5. Ortega-Ferrusola, C.; Sotillo-Galan, Y.; Varela-Fernandez, E.; Gallardo-Bolanos, J.M.; Muriel, A.; Gonzalez-Fernandez, L.; Tapia, J.A.; Pena, F.J. Detection of “apoptosis-like” changes during the cryopreservation process in equine sperm. *J. Androl.* **2007**, *29*, 213–221.

6. Guthrie, H.D.; Welch, G.R. Determination of intracellular reactive oxygen species and high mitochondrial membrane potential in Percoll-treated viable boar sperm using fluorescence-activated flow cytometry. *J. Anim. Sci.* **2006**, *84*, 2089–2100.

7. Nagy, S.; Jansen, J.; Topper, E.K.; Gadella, B.M. A Triple-Stain Flow Cytometric Method to Assess Plasma- and Acrosome-Membrane Integrity of Cryopreserved Bovine Sperm Immediately after Thawing in Presence of Egg-Yolk Particles. *Biol. Reprod.* **2003**, *68*, 1828–1835.

8. Mortimer, D.; Curtis, E.F.; Miller, R.G. Specific labelling by peanut agglutinin of the outer acrosomal membrane of the human spermatozoon. *J. Reprod. Fertil.* **1987**, *81*, 127–135.

9. Harrison, R.A.P.; Mairet, B.; Miller, N.G.A. Flow cytometric studies of bicarbonate-mediated Ca2+ influx in boar sperm populations. *Mol. Reprod. Dev.* **1993**, *35*, 197–208.

10. Takahashi, A.; Camacho, P.; Lechleiter, J.D.; Herman, B. Measurement of Intracellular Calcium. *Physiol. Rev.* **1999**, *79*, 1089–1125.

11. Yeste, M.; Fernández-Novell, J.M.; Ramió-Lluch, L.; Estrada, E.; Rocha, L.G.; Cebrián-Pérez, J.A.; Muiño-Blanco, T.; Concha, I.I.; Ramírez, A.; Rodríguez-Gil, J.E. Intracellular calcium movements of boar spermatozoa during “in vitro” capacitation and subsequent acrosome exocytosis follow a multiple-storage place, extracellular calcium-dependent model. *Andrology* **2015**, *3*, 729–747.

12. Hayato, R.; Higure, Y.; Kuba, M.; Nagai, H.; Yamashita, H.; Kuba, K. β3-Adrenergic activation of sequential Ca2+ release from mitochondria and the endoplasmic reticulum and the subsequent Ca2+ entry in rodent brown adipocytes. *Cell Calcium* **2011**, *49*, 400–414.

13. De la Fuente, S.; Fonteriz, R.I.; Montero, M.; Alvarez, J. Dynamics of mitochondrial [Ca2+] measured with the low-Ca2+-affinity dye rhod-5N. *Cell Calcium* **2012**, *51*,

65–71.
